# Blunt renal trauma in children: the experience of Mohammed VI University Hospital of Oujda in Morocco between 2015 and 2021

**DOI:** 10.11604/pamj.2022.41.347.31945

**Published:** 2022-04-29

**Authors:** Abdelouhab Ammor, Kamal El Haissoufi, Mariame Karrouchi, Siham Nasri, Imane Skiker, Houssain Benhaddou

**Affiliations:** 1 Department of Uro-Visceral and Genital Paediatric Surgery, Mohammed VI University Hospital, Oujda, Morocco,; 2 Faculty of Medicine and Pharmacy, Mohammed I University, Oujda, Morocco,; 3 Department of Radiology, Mohammed VI University Hospital, Oujda, Morocco

**Keywords:** Blunt renal trauma, American Association for the Surgery of Trauma (AAST), Kidney Injury Scale, conservative management, children

## Abstract

**Introduction:**

blunt renal traumas in children are rare and their management is not suited to a very clear consensus. We sought to report our experience in managing renal injuries in children presented after blunt abdominal trauma.

**Methods:**

data of children aged less than 16 years with blunt renal injuries between January 2015 and April 2021 were retrospectively reviewed. Demographic characteristics, clinical course, biological results, radiological findings, associated injuries, management and follow up of included patients were described. Renal lesions were classified according to the American Association for the Surgery of Trauma (AAST).

**Results:**

we included a total of 20 children, of whom 70% (n=14) were males. The mean of age was 8.50 ± 3.42 years. Falls in 65% (n=13) and motor-vehicle accidents in 35% (n=7) were the two main mechanisms of injuries. Abdominal pain was the most common symptom and macroscopic hematuria was assessed in 55% of patients (n=11). Low-grade injuries (I-III) represented 40% of the cases (n=8), 60% of injuries were AAST grade IV (n=12) and none with AAST grade V was diagnosed. Spleen injuries in 25% (n=5) as well as traumatic brain injuries in 25% (n=5) were the most identified concomitant injuries followed by liver lesions in 15% (n=3). 75% of renal injuries (n=15) were managed conservatively and all cases that required an operative management were with AAST grade IV. No nephrectomy in our series was performed and the follow up was favorable with a median of 3 years.

**Conclusion:**

our data suggest that the majority of children with blunt renal injuries can be managed conservatively regardless the grade of lesions as long as no hemodynamic instability or symptomatic urinoma are identified.

## Introduction

Traumatic injuries within pediatric population are a major cause of mortality and the most common lesions occurred after blunt trauma [1]. Children are more likely to present renal injuries than adults after abdominal blunt trauma [2]. However, blunt renal injuries account for 8 to 12% of all abdominal blunt lesions in children and are less common than those of spleen and liver [1,3]. The purpose of management strategy of blunt renal injury is to preserve renal function as long as possible [1]. Therefore, multiple management options were evaluated but no very clear guidelines were established to reach this goal [1]. The aim of our study was to report our experience in the management of blunt renal injuries in children in an academic center for a period of 6 years.

## Methods

**Study design and setting:** this is a retrospective single-centred observational study that was carried out at the Department of Uro-visceral and Genital Pediatric Surgery attached to Mohammed VI University Hospital in Oujda, Morocco. This hospital serves the Oriental Region of Morocco, which covers an area of over 90130 km^2^ or 12.7% of the national territory. All children with blunt renal trauma admitted to the hospital through its Emergency Department are discussed at the daily medical meeting consisting of urologic pediatric surgeons, professors and resident doctors.

**Study population:** children aged less than 16 years old with a diagnosis of blunt renal trauma between January 2015 and April 2021 in our academic institution were evaluated. Based on CT findings and according to the American Association for the Surgery of Trauma (AAST) Kidney Injury Scale, the renal lesions in our patients were classified on a scale of 1 to 5 (Table 1) [4]. Depending on hemodynamic stability and associated injuries of other organs, a conservative managment was adopted for all patients in whom surgery was not necessary. Surgical procedures were indicated in patients with hemodynamic instability (arterial hypotension, tachycardia, continuous decrease of hematocrit values after fluid resuscitation and blood transfusions). Otherwise, strictly bed rest, pain evaluation and managment, a monitoring of vital parameters and hematocrit represented the standard of care. A urethral Foley catheter was placed in patients who initially presented hematuria to follow its evolution objectively. After hospital discharge, a long term evaluation of renal injuries was performed for all patients by Doppler ultrasound.

**Table 1:** AAST Kidney Injury Scale (Reprinted from Journal of the American College of Surgeons. Vol 207. Glen Tinkoff, Thomas J. Esposito, James Reed, Patrick Kilgo, JohnFildes, Michael Pasquale, J. Wayne Meredith. American Association for the Surgery of Trauma Organ Injury Scale I: Spleen, Liver, and Kidney, Validation Based on the National Trauma Data Bank. Pages 646 to 655. Copyright (2008), with permission from Elsevier)

Grade	Injury type	Description of injury
I	Contusion Hematoma	Microscopic or gross hematuria, urologic studies normal Subcapsular, nonexpanding without parenchymal laceration
II	Hematoma Laceration	Nonexpanding perirenal hematoma confirmed to renal retroperitoneum <1.0 cm parenchymal depth of renal cortex without urinary extravasation
III	Laceration	>1.0 cm parenchymal depth of renal cortex without collecting system rupture or urinary extravagation
IV	Laceration Vascular	Parenchymal laceration extending through renal cortex, medulla, and collecting system Main renal artery or vein injury with contained hemorrhage
V	Laceration Vascular	Completely shattered kidney Avulsion of renal hilum that devascularizes kidney

**Data collection:** this study is based on patients data collected from their medical records. Age, gender, mechanism of injury, injury localisation, initial symptoms and clinical findings, biological parameters, abdominal US and/or CT results, concomitant injuries of other organs, management, length of stay and follow up of included children were specified.

**Data management and analysis:** the collected data were captured onto Microsoft Excel and data analysis was performed by using SPSS 26.0. Number of patients was defined as (n) and percentage in all data was defined as (%).

## Results

**General characteristics:** we included a total of 20 children aged less than 16 years old. The median of age was 8.50 years (IQR: 6.25-11). 70% (n= 14) of the patients in this group were male and 30% (n = 6) were female. Falls represented the most common mechanism of injuries in 65% (n= 13) followed by motor-vehicle accidents in 35% (n= 7). 35% (n= 7) of the falls were from high levels and 30% (n= 6) were from standing height.

**Figure 1:**
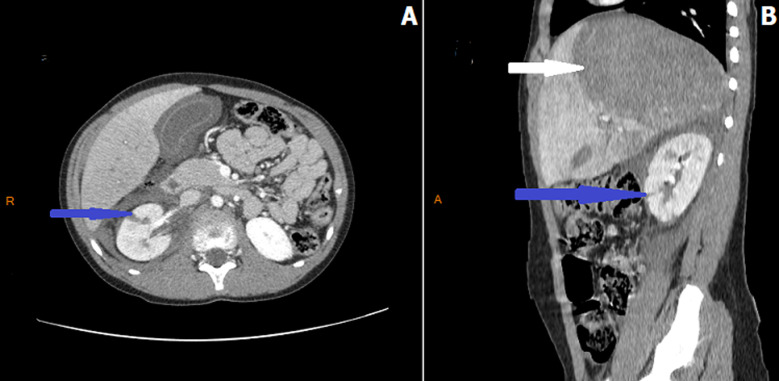
A) axial and B) sagittal abdominal contrast enhanced CT performed after a blunt abdominal trauma in an 11-years-old boy showing a parenchymal laceration of the right kidney (grade III of AAST Kidney Injury Scale) (blue arrows) associated with a voluminous hematoma of the liver (white arrow) after falling from standing height and who was managed conservatively

**Figure 2:**
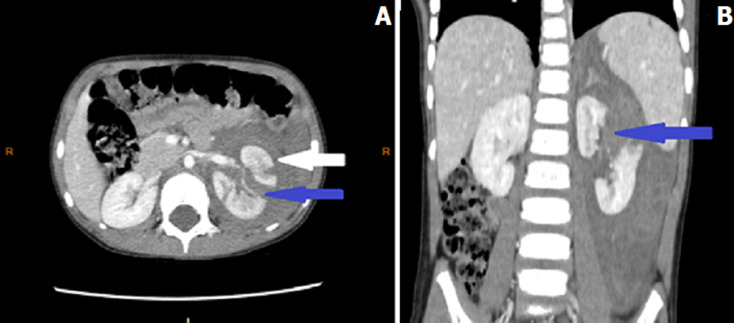
A) axial and B) coronal abdominal contrast enhanced CT performed after a blunt abdominal trauma in a 2-years-old boy showing an extended parenchymal laceration of the left kidney (grade IV of AAST Kidney Injury Scale) (blue arrows) associated with perirenal hematoma (white arrow) after falling from high and who was managed conservatively

**Clinical presentation:** abdominal pain was the most common sign and was present in 18 patients (90%). For the 2 other children, pain was difficult to identify due to the state of unconsciousness (10%). Importantly, hematuria was only assessed in 11 children (55%) (Table 2). The right kidney was the most concerned in 60% (n= 12) and no case of bilateral renal trauma in this series was described. Abdominal computed tomography (CT) was performed in all cases and abdominal ultrasound (US) was done only for 11 patients (55%) as first radiological examination. Basing on CT results, 1 patient had grade I injuries (5%), 3 had grade II injuries (15%), 4 with grade III injuries (20%), 12 had grade IV injuries (60%) and no case with grade V injuries in our series was identified (Figure 1 and Figure 2). Within patients with grade IV injuries, 2 had renal pseudoaneurysm (10%). Moreover, spleen injuries were diagnosed in 5 patients (25%) as well as traumatic brain injuries (25%) representing the most common concomitant lesions followed by those of the liver in 3 patients (15%). There were injuries of adrenal and pancraes in only 2 patients (10%).

**Table 2:** assessment of hematuria and blood transfusion requirement according to the grade of renal injury

Grade (AAST)	Hematuria			Blood transfusion		
	Number of cases	n(%)	N (%)	Number of cases	n (%)	N (%)
I	0/1	0	0	0/1	0	0
II	0/3	0	0	1/3	33.3	5
III	2/4	50	10	1/4	25	5
IV	9/12	75	45	4/12	33.3	20
V	0/0	0	0	0/0	0	0

**AAST:**American Association for the Surgery of Trauma; **N :**general percentage; **n :**percentage within grade group

**Management and outcomes:** the need of blood transfusion was required only for 6 patients (30%) (Table 2). Within the 20 patients, an operative management for the kidney was necessary only for 5 cases (25%). Remarkably, all the five cases had grade IV renal injuries. Surgery was required for two children (10%) because of hemodynamic instability despite repetitive blood transfusions and an hemostatic kidney repair was performed. Additionally, two cases (10%) with renal pseudoaneurysm was managed operatively; an angiographic embolization was successfully performed for one and the other was managed surgically where an embolization was not accessible. The fifth case presented a urinoma and a stent was placed with a good evolution (Table 3). In addition, the mean hospital length of stay (LOS) was 12 days where the shortest hospital stay was 5 days and the longest was 42 days. All patients were discharged from hospital in a good general condition and no complications such as impairment in renal function, hypertension or arteriovenous fistula were noted with a median follow up of 3 years. Relevant characteristics of included children are summarized in Table 4.

**Table 3:** operative management in our case series and its requirement according to the AAST Kidney Injury Scale

Grade (AAST)	Number of cases / cases with need for operative management	Operative management			
		Stent	Kidney repair	Vascular ligature	Embolization
I	1/0	0	0	0	0
II	3/0	0	0	0	0
III	4/0	0	0	0	0
IV	12/5	1	2	1	1
V	0/0	0	0	0	0
Total	20/5	1	2	1	1

**AAST:** American Association for the Surgery of Trauma

**Table 4:** a summary of relevant characteristics of the 20 evaluated children

Grade (AAST)	Age (years)			Gender		MOI			Injured kidney		Mean of LOS in days (range)	Associated injuries (N)	Total
	1-6	7-11	12-16	M	F	FSH	FFH	MVA	Right	Left			
I	0	1	0	1	0	0	0	1	0	1	8	Traumatic brain injury (1) Liver injury (1) Leg fracture (1)	1 (5%)
II	0	3	0	3	0	0	3	0	1	2	11.67(11-12)	Traumatic brain injury (1) Hemo/ pneumothorax (2) Splenic injury (2) Pancreatic injury (1)	3(15%)
III	0	3	1	3	1	1	0	3	4	0	7.75 (5-11)	Traumatic brain injury (1) Splenic injury (1) Liver injury (2) Isolated kidney (1)	4 (20%)
IV	5	6	1	7	5	5	4	3	7	5	13.83 (5-42)	Traumatic brain injury (2) Hemo/ pneumothorax (2) Splenic injury (2) Adrenal haematoma (1) Isolated kidney (10)	12 (60%)
V	0	0	0	0	0	0	0	0	0	0	0	0	0 (0%)

**AAST:** American Association for the Surgery of Trauma. **M:** male. **F:** female. **MOI:** mechanism of injury. **FSH:** fall from standing height. **FHL:** fall from high level. **MVA:** motor vehicle accident. **N:** number of patients. **LOS:** length of stay

## Discussion

Blunt renal injuries in children are uncommonly seen within all pediatric blunt traumas with an estimated incidence of only 1.2% [5]. Five to twenty percent (5%-20%) of children with blunt abdominal trauma (BAT) are susceptible to have a lesion of the kidney [6]. Among children with abdominal solid organ injuries, renal lesions are less frequently detected than those of liver and spleen [3]. Importantly, blunt traumas were reported to be the most common mechanism of renal injuries in children [6,7]. In fact, multiple factors explaining why children are more predisposed to renal injuries after an abdominal blunt trauma than adults were cited: relative increased renal size, flexibility and thinness of anatomic surrounding structures (perirenal fat, Gerota´s fascia and the capsule) and incomplete ossification of lower ribs [6]. Through the present study, we sought to report the findings of our experience in the management of blunt renal trauma in children.

The mean age of children with blunt renal trauma (BRT) was reported to be more than 10 years with a predominance of male gender [4,5]. Rapid decelerations including falls from heights and motor-vehicle accidents, and direct hits to the flank were more likely described as potential mechanisms of renal injuries in blunt traumas [8]. As clinical manifestations, abdominal or pelvic pain, complaints of flank, flank ecchymosis, hematuria or a fracture of distal ribs were cited as revelation signs of possible injured kidney in children [9]. Multiple studies were achieved and reported in the literature to discuss the utility of microscopic hematuria (MH) in predicting renal lesions or their severity after BAT in children, but their results seem to be controversial. Holmes JF *et al*. concluded to an independently significant correlation between the presence of MH on urinanalysis (UA) (>5 red blood cells per high powered field (RBCs/HPF)) and intra-abdominal lesions in children after BAT [10]. Importantly, DJ Isaacman *et al*. reported a sensitivity of 100% with a negative predictive value (NPV) of 100% of MH (>5 RBCs/HPF) for the detection of intra-abdominal injury in children when combined with the physical examination findings [11]. Interestingly, GA Taylor *et al*. found none of 41 children with asymptomatic MH evaluated prospectively after BAT had an abdominal injury on CT imaging and, thereby, the presence and the severity of hematuria can be considered as useful predictive markers for intra-abdominal injury only when other suggestive symptoms are associated [12]. Additionally, a disagreement in the literature is noticed in term of studying the correlation between the severity of renal injury and the hematuria degree. According to HP Stalker *et al*., a direct relationship between the renal injury severity and hematuria amount were found and normotensive children with <50 RBCs/HPF had no significant renal lesions while all children with severe grade renal injury were schocked or had an amount of hematuria >99 RBCs/HPF [13]. In contrast, a recent study showed that many children with large hematuria had no renal injury and no significant association between degree of hematuria and renal lesions was found [14]. In our series, only macroscopic hematuria was assessed and, if absent, did not rule out renal lesions even in patients with high-grade injuries (grade IV).

Radiologically, abdominal US and CT are the two must imaging exams that have important accuracy in detecting post-traumatic renal lesions in children [15]. US can mainly be performed in stable patients with isolated and low-energy abdominal trauma. Its diagnostic accuracy is considerably improved after resorting to contrast-enhanced ultrasonography (CEUS) which presents a higher sensitivity in the detection of renal parenchymal lesions and peri-renal hematoma. Additionally, abdominal enhanced CT scan is the exam of choice for the evaluation of renal injuries and is especially indicated in patients with high-energy trauma and in whom a hemodynamic stability is ensured [15]. Notably, US has a sensitivity of 79-100% with a NPV of 97-100% in detecting grade III-V renal injuries in children when compared to the findings of enhanced CT scan as the gold standard [16]. However, the US performance seems to be poor to diagnose or rule out low-grade renal injuries [16]. We have used CT in the diagnosis and in grading of renal trauma in all patients of our series. In pediatric population, the conservative approach is the best choice in the management of blunt renal trauma as long as the child is hemodynamically stable [6]. Indeed, all grades I to III require a nonoperative management when hemodynamic stability is achieved [17]. For grade I injuries, the patients don´t even require an hospitalization and can be discharged home immediately as long as no gross hematuria exists. However, grade II and III patients should be admitted in hospital for at least 24 hours considering the risk of bleeding that majorely occurs during the first day after trauma, and to reinforce the psychological status of the child and his family in term of injury seriousness. In contrast of low-grade renal injuries, an operative management can be necessary in 59% of cases of grade IV and for the most patients with grade V as a significant bleeding threatening life is associated [17]. In a systematic review, this pourcentage was reported to be lower (28%) and nonoperative management was very recommended in the treatment of grade IV renal lesions if there is no hemodynamic instability or symptomatic urinoma [18]. In our series, all the patients were hospitalized including those with grade I, five children with grade IV (41%) required an operative management and no nephrectomy was done. Otherwise, no case with grade V was identified and all patients with low-grade renal injuries were managed conservatively.

In terms of complications after blunt renal trauma in children, functional outcome of the injured kidney by radionuclide study after healing seems to be correlated with injury grade [19]. In addition, a low risk of developing hypertension was reported after high-grade blunt renal trauma and that only during the immediate post-trauma period [20]. In our series, no functional radionuclide study was done because of its unavailability in our institution. Nevertheless, a Doppler ultrasound was performed and a radiological complete healing of the kidney was assessed in all the cases of our series. Furthermore, no case of hypertension was diagnosed whether at the acute period or during the long-term follow-up. There are some limitations in this study. First, the overall low number of cases included due to the rareness of the blunt renal trauma in childhood. Second, the descriptive nature of the study explained by the small sample size not allowing getting reliable analytic results between subgroups. Finally, it is a retrospective single-center study leaded in an academic center. Therefore, further prospective cohorts with large sample sizes are required in order to discuss critically our findings.

## Conclusion

To sum up, our work has led us to conclude that renal injuries are uncommonly seen in children presented with blunt abdominal trauma. Abdominal pain and hematuria are the most frequent clinical symptoms of revelation. Abdominal enhanced CT scan is the gold standard for the evaluation of renal injuries in children. Children with blunt renal injuries can be managed conservatively regardless the grade of lesions as long as no hemodynamic instability or symptomatic urinoma are identified.

### What is known about this topic

- Blunt renal traumas in children are rare;
- The management of blunt renal trauma in children is not suited to a very clear consensus.

### What this study adds

- Blunt renal injuries are rarely seen in childhood;
- Macroscopic hematuria is not a stable sign to reveal a renal injury in children even for high grade lesions;
- The conservative management is a reliable and efficient approach even for high-grade renal injuries.
